# Developing a guide for sustainable healthcare practice: A case study from the Swedish Society of Medicine

**DOI:** 10.1016/j.joclim.2025.100413

**Published:** 2025-01-11

**Authors:** Andreas Vilhelmsson, Ida Persson Cofina, Maria Wolodarski, Tobias Alfvén

**Affiliations:** a Lund University, Department of Laboratory Medicine, Division of Occupational and Environmental Medicine, Sweden; b Lund University, Department of Clinical Sciences Malmö, Center for Primary Health Care Research. Region Skåne, Primary health care, Sweden; c Karolinska University Hospital Department of Oncology and Pathology, Karolinska Institutet, Stockholm, Sweden; d Department of Global Public Health, Karolinska Institutet, Stockholm, Sweden; e Sachs’ children and youth hospital, Stockholm, Sweden

**Keywords:** Climate change, Public health, Sustainability, Greener healthcare, Environment, Co-benefits

## Abstract

**Introduction:**

Although climate change has been consistently identified as one of the greatest threats to humans, many clinicians do not feel prepared to address climate change with patients and medical school curricula still have very little coverage of its health consequences. At the same time, health care providers have been shown to be trusted voices and are therefore well suited to help build the public and political necessary to enact policies that effectively address climate change and protect human health in equitable ways. Nevertheless, teaching climate, health and sustainable healthcare to medical colleagues, students and healthcare leaders can be challenging for various reasons. It is therefore essential to provide health care providers and leaders with the appropriate tools and communication skills to facilitate a more sustainable and greener healthcare.

**Case presentation:**

In this case report, we describe how we developed a sustainability guide for clinicians with examples of how to reduce unnecessary environmental and climate impact, without compromising patient safety, highlighting potential co-benefits for public health, healthcare efficiency, financial aspects and to the occupational environment.

**Discussion:**

The sustainability guide has been appreciated by medical specialties as a tool to illustrate concrete ways of working with sustainable healthcare in Sweden. It has also been used to introduce the field into the medical curriculum at Swedish universities and teach students in sustainability.

**Conclusion:**

Our sustainability guide highlights the potential value of providing health care personnel and leaders with the appropriate tools and communication skills to facilitate a more sustainable and green healthcare.

## 1 Introduction

Climate change represents one of the greatest global health threats of the 21st century [1]. It is already adversely affecting human health and threatens to disrupt health care systems’ ability to deliver high-quality care [2,3]. Climate change risks undermining the past 50 years of gains in public health due to more extreme weather events such as intense heat waves, flooding and damaging storms, as well as a changing pattern of emerging infectious diseases [4]. At the same time, healthcare is a contributor to climate change through its resource-demanding and polluting activities [5]. Globally, it is estimated that the healthcare sector accounts for approximately 4–6% of global greenhouse gas (GHG) emissions [1], with large variations among countries. For example, the GHG share of the healthcare system in the United States is 8.5% [6], whereas in Sweden healthcare accounts for 4.4% of total emissions [7]. Thus, the healthcare sector has a major responsibility when it comes to climate change adaptation and mitigation. Moreover, it constitutes a unique platform to provide meaningful climate action. Health care providers have been shown to be trusted voices [8] and are therefore well suited to help build the public and political will necessary to enact policies that effectively can address climate change and protect human health in equitable ways [9]. Still, healthcare professionals in general do not feel prepared to address climate change with patients [10] and medical school curricula have insufficient education regarding the health consequences of climate change [11]. In Sweden, as in many other countries, there are still inadequate official education efforts for medical students and health providers regarding climate change and health, despite Swedish law requiring sustainability aspects to be integrated into all higher education [12].

## 2 Case presentation

### 2.1 The decision to develop a sustainability guide

In this paper, we present a case-study of developing a guideline aimed to engage health care professionals in working towards more sustainable healthcare.

Discussions with healthcare leaders, strategists and decision-makers, identified the need to illustrate the importance of concepts, such as ecological sustainability. Moreover, while wanting to contribute to more sustainable healthcare, many clinicians asked not only what they could do as professionals, but why they should change their way of working. There was also a call for concrete and practical solutions from medical professionals.

**Fig. 1:**
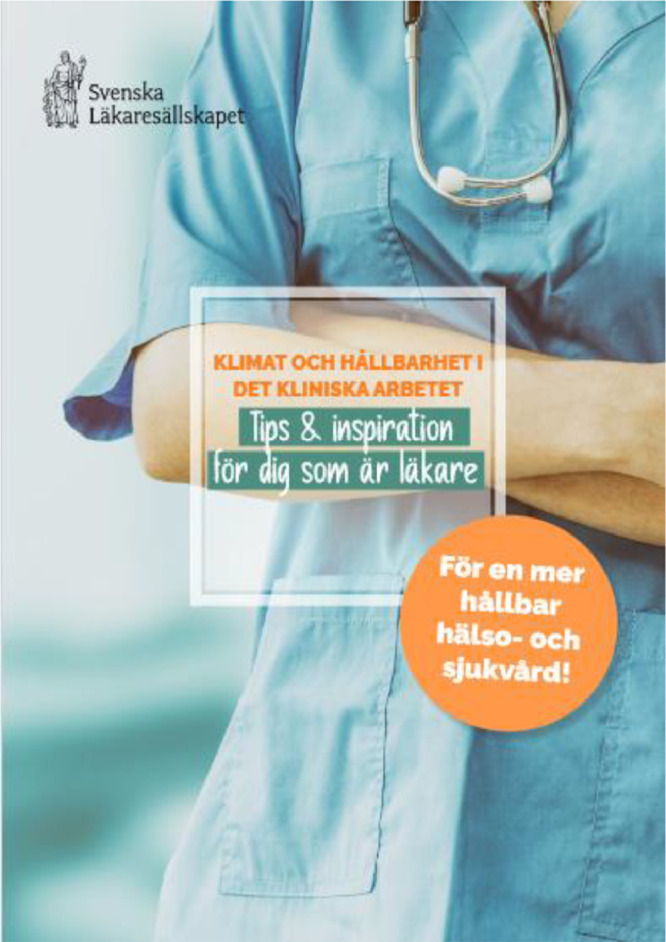
The cover to the sustainability guide from the Swedish Society of Medicine. The Swedish original title of the guide translates to something like 'Climate and sustainability in clinical practice: Tips and inspiration for you as a physician’.

The decision to develop a sustainability guide began in 2019 when the Swedish Society of Medicine (SLS) working group for climate, health and sustainable healthcare was appointed by the SLS to develop a climate policy with the aim of contributing to reduced climate impact and a more sustainable healthcare [13]. The work was initially performed and published as a quality improvement project within general medicine in a Swedish region but was subsequently adjusted and broadened to be applicable to most specialties. It was launched at the Swedish Society of Medicine's Berzelius symposium on the theme of planetary health in 2022 and was distributed in the SLS's web channels, through the Swedish Medical Magazine (Läkartidningen) and regional channels (see Fig. 1).

## 3 Principles for a sustainable health system

Three general principles on healthcare sustainability from the Lancet planetary health framework served as a template when the guide was developed [14]:

### 3.1 Principle 1: health promotion and disease prevention

In the first principle, examples of how to discuss behavioral habits, especially dietary recommendations and active transport, were listed. The co-benefits between health-promoting activities for patients, public health and planetary boundaries were emphasized [15]. Dietary recommendations were based on the Nordic Nutrition Recommendations which also emphasize the environmental aspects of healthy diets [16].

### 3.2 Principle 2: to avoid excessive and unnecessary investigation, intervention and treatment

This principle was exemplified by medication prescriptions, resource stewardship with materials and devices, continuity amongst caregivers to optimize care and through the promotion of virtual follow-ups when considered safe and applicable. These examples emphasized that ecological, social and financial considerations often interact with each other and that there are further positive synergies when time consumption and the work environment are also considered. Striving for continuity of care, where a patient meets the same health professional or team over time, constitutes an example that has been shown to increase resource stewardship while increasing patient satisfaction and possibly also patient safety [17]. Healthcare sustainability also involves identifying actions that have synergies so as to provide “added value”. Thus, the second principle is in line with the Choosing Wisely Campaign, a global initiative for sustainable healthcare [18], to which Sweden has been affiliated since 2024 through its version “Kloka Kliniska Val” (“Wise Clinical Choices”) [19].

### 3.3 Principle 3: reducing the environmental and carbon footprints of health services and products (“greening of the healthcare sector”)

The third principle focused on the lion's share of the healthcare climate footprint: mitigating the emissions from the supply chain, through streamlining health services, the principles to avoid, reduce, reuse, recycle [20] and by choosing low-carbon alternatives. The focus here was on healthcare materials/instruments/textiles/devices and on sustainable drug prescriptions, such as non-pharmacological alternatives, systematic medical follow-ups, start packages, disposal of medical waste and selection of the least harmful alternatives. The Swedish Strategic Programme against Antibiotic Resistance (Strama), [21] was highlighted as a role model for improving patient health and the health of future generations and for reducing pollution from broad spectrum antibiotics in nature. Other important pharmacological recommendations concerned avoiding medicines, such as fluoroquinolones because of their resistance in nature and instead of metered dose inhalers prescribe dry powder inhalers to reduce unnecessary release of greenhouse gases [22]. Multi-use devices and textiles were recommended instead of single use to reduce greenhouse gas emissions and waste production [23].

### 3.4 Streamlining health services

The sustainability guide also calls for efforts to streamline the patient journey by coordinating healthcare services. Virtual follow-up meetings constitute an example that can save both time and transport for patients and healthcare staff while also reducing unnecessary emissions. Notably, there is a natural overlap between the second and the third principles regarding sustainable material use and drug prescription. Excessive and unnecessary tests and treatments should be avoided, and low-carbon/low-environmental impact alternatives need to accompany each other. The same is true for streamlined healthcare services.

### 3.5 The art of climate communication

The last focus of the sustainability guide concerned communication and the need for clinicians to commit to health advocacy at all levels of society. This is in line with both behavioral models of acting for impact and with the position of the World Health Organization (WHO) [24] and the World Medical Association (WMA) [25]. Health professionals are encouraged to demand ecological considerations both in and out of the clinical setting, for instance of healthcare leaders, politicians and other healthcare providers, including those in the pharmaceutical industry and medical device companies [8,26]. According to behavioral models on ecological sustainability, working at higher levels of impact (such as organizational, public and cultural levels), might be of greater importance than individual actions in the private or professional spheres. These findings indicate that communication is a crucial part of the transition process and must accompany clinical actions [27]. For example, avoiding medicines like fluoroquinolones because of their resistance in nature, might be beneficial to the environment, but to increase impact, this action should be communicated to colleagues and to the public to inspire others and form new norms.

## 4 Discussion

The sustainability guide provide physicians and medical students in Sweden with a toolkit to teach and communicate climate, health and sustainable healthcare. The communication literature is constantly evolving and toolkits and guides for various specialties are being produced, both as national initiatives and as international consensus documents. For example, the WHO recently released a toolkit designed to equip healthcare workers with the knowledge and confidence to communicate effectively about climate change and health [27,28]. Additionally, guidelines for anesthesiologists [29], guidelines and tools for a sustainable and decarbonized health care [30] and toolkits for a smart hospital [31] have been presented. Some more general educational platforms and guidelines have also been published, together with documents on how to introduce and implement these aspects into the medical curriculum [32,33].

What these guidelines and toolkits lack however is to specifically give healthcare providers advice on streamlining healthcare (points 3.2 and 3.4 above) or “greener” medication choices ( point 3.3 above). To our knowledge, our guideline is the first to do just that.

To date, the sustainability guide has been appreciated by many medical specialties as a tool to illustrate concrete ways of working with sustainable healthcare in Sweden. Clinicians can find relevant parts of the guide and develop an understanding of ways they can personally contribute to healthcare sustainability. After publication the working group received several invitations to present the guide to medical colleagues at various clinics and to regional environmental strategists. The guide has also been used to introduce the field into the medical curriculum at Swedish universities to teach students in sustainability. For example, the guide has been used in medical education as a tool to enhance sustainability in fictive patient scenarios. Certain sections of the guide may also be relevant to other health professions as well. As an example, the guide directly inspired the Swedish Nursing Association to develop their own guide [34]. Furthermore, associations of physiotherapists, nutritionists and pharmacists were also appreciative of the guide.

The guide emphasizes that patient safety is always a top priority and that the focus lies on clinical activities benefitting all aspects of sustainability (efficacy, financial, occupational environment, time consumption and so on), contrary to what is sometimes argued when sustainability and environmental aspects within healthcare are being discussed.

Despite various efforts to promote the guide, its impact has not yet been measured among clinical users. Nevertheless, it has been introduced as a mandatory part of the medical curriculum at some Swedish universities and examination results indicate that medical students gain new knowledge and skills and find it useful. Conducted seminars and written examinations indicate that students now have acquired both knowledge and skills to enhance ecological sustainability in the clinical setting.

Limitations and areas for further improvement of the guide include the fact that generalizing and exemplifying sustainability might undermine the complexity of various fields. Highlighting some concerns may also cause others to be ignored, such as health equity, which is not sufficiently discussed in the guide. Climate risks are also unevenly distributed, and both create new health inequities and exacerbate existing inequities [35]. Furthermore, not all patients will follow “lifestyle recommendations”, and it is important not to discriminate against these individuals. Still, prevention and health promotion are ways to increase equity within healthcare. Studies also suggest that from a public health perspective, upstream strategies such as policymaking and mandatory interventions may be more beneficial in achieving results [36] and related education should accompany the clinical recommendations of the guide.

The carbon footprint from medical devices and pharmaceuticals also varies depending on local circumstances, such as energy sources and methods used in production, transport and disposal. Progress is being made both in assessing the carbon footprint of healthcare settings [37] and the efforts to reduce it. For example, metered dose inhalers with a significantly lower climate impact than previous alternatives are being developed, possibly calling for updated recommendations [38].

An updated version of the sustainability guide that considers new research, guidelines and health equity is being planned. The revised sustainability guide will use an interdisciplinary approach and be translated into English so it can be used in various educational settings. Other aspects of sustainability will include patient self-care, shared decision making (in line with the Centre for Sustainable Healthcare) [39] and healthcare resilience strategies to address extreme weather and conflicts, such as protecting vulnerable groups from the health risks of climate change and actions to secure a robust healthcare system.

## 5 Conclusion

With increasing climate impacts on health care systems, the need for mitigation and adaptation has increased. By developing a sustainability guide for clinicians with examples of ways to reduce unnecessary environmental and climate impacts without compromising patient safety, we hope to offer physicians and medical students knowledge and practical skills to facilitate a transition to more sustainable and greener healthcare.

## Funding

This research did not receive any specific grant from funding agencies in the public, commercial, or not-for-profit sectors.

## CRediT authorship contribution statement

**Andreas Vilhelmsson:** Writing – review & editing, Writing – original draft, Conceptualization. **Ida Persson Cofina:** Writing – review & editing, Writing – original draft, Resources, Project administration, Investigation, Conceptualization. **Maria Wolodarski:** Writing – review & editing, Project administration. **Tobias Alfvén:** Writing – review & editing.

## Declaration of competing interest

The authors declare that they have no known competing financial interests or personal relationships that could have appeared to influence the work reported in this paper.
